# *Cistanches deserticola* PhG-RE through Inhibiting ERS Apoptosis Mechanism to Protect Myocardial Cell Apoptosis from H_2_O_2_-Induced Endoplasmic Reticulum Stress

**DOI:** 10.1155/2020/8219296

**Published:** 2020-09-25

**Authors:** Tianwei Lan, Qian Yu

**Affiliations:** Department of Pharmacy, China-Japan Union Hospital of Jilin University, Changchun 130033, China

## Abstract

The herb *Cistanche deserticola* has some myocardial protective effects. This study attempted to explain the mechanism by which PhG-RE protects myocardial cells and verify if this protection occurs through regulating the apoptosis mechanism associated with endoplasmic reticulum stress (ERS). Rat myocardial cells were exposed to 150 *μ*g·mL^−1^ PhG-RE for 24 h and then to 100 *μ*mol·mL^−1^ H_2_O_2_ for 18 h to induce ERS and establish a cell damage model. Thapsigargin (TG), a specific ERS activator, and 4-phenylbutyric acid (4-PBA), an ERS inhibitor, were used to validate the accuracy of the experiment. Our results demonstrated that PhG-RE significantly improved cell viability, protected cells, and reduced cell damage and apoptosis. PhG-RE played a role similar to that of the ERS inhibitor 4-PBA in protecting myocardial cells against apoptosis and damage induced by ER stress. Furthermore, PhG-RE significantly attenuated the mRNA expression of the ERS-associated apoptotic factors GRP78, CHOP, and Caspase-12 and the protein expression of the ERS-associated apoptotic factors GRP78, CHOP, Caspase-12, and p-JNK. Taken together, these findings suggest that PhG-RE can effectively protect myocardial cells and reduce cell apoptosis and damage, which may be related to the regulation of ERS-associated apoptosis.

## 1. Introduction

*Cistanche deserticola* (*C. deserticola*) is one of the herbal plants used in traditional Chinese medicine that grows in desert environments. The phenylethanoid glycoside-rich extract (PhG-RE) is the main active component of *C. deserticola*. Research has found that PhG-RE can protect the myocardium against ischaemia-reperfusion (I/R) injury [1].

When ERS starts to occur in myocardial cells, the level of glucose regulated protein 78 (GRP78) increases to antagonise an ERS-induced injury. As ERS progresses, apoptosis is initiated, and the expression of CCAAT/enhancer-binding protein homologous protein (CHOP), c-Jun N-terminal kinase (JNK), and cysteinyl aspartate specific proteinase-12 (Caspase-12) increases. For example, the expression of GRP78, CHOP, and cleaved ATF6 (c-ATF6) in myocardial cells of rats with HF is significantly increased, and PERK (p-PERK), IRE1 (p-IRE1), and p-JNK are activated [2]. In addition, ERS induced by I/R injury increases the expression of GRP78 and CHOP [3]. The phosphorylation levels of PERK and eIF2*α* are increased [4]. The expression of pro-apoptotic proteins Bax and Caspase-3 are increased, causing cell rupture and myocardial remodelling [5], and the area of myocardial infarction and myocardial enzymatic activity is increased [3]. It was found during our previous research that PhG-RE can protect the myocardium against ischaemia-reperfusion injury and can significantly reduce the area of myocardial infarction induced by I/R [1]. However, it is unclear whether PhG-RE can inhibit ERS-associated apoptosis and thereby reduce myocardial cell loss and apoptosis.

In this study, cells were pretreated with PhG-RE to observe whether PhG-RE can inhibit H_2_O_2_-induced ERS and therefore reduce myocardial cell loss and apoptosis. Thapsigargin (TG), a specific ERS activator, and 4-phenylbutyric acid (4-PBA), an ERS inhibitor, were used to further verify whether mediating ERS can effectively reduce cell apoptosis and whether this process is related to the expression levels of GRP78, CHOP, JNK, and Caspase-12, which have been shown to mediate ERS-associated apoptosis.

## 2. Materials and Methods

### 2.1. Reagents

The materials and methods section should be exhaustive, so that all procedures can be repeated. It may be divided into headed subsections if several methods are described. H9c2 rat myocardial cells were obtained from the Cell Bank of the Chinese Academy of Sciences (Shanghai, China). *C. deserticola Cistanche deserticola* PhG-RE was obtained from Changchun Medicinal Material Co. (Changchun, China), identified by Dr Zhong-ying Liu (School of Pharmaceutical Sciences, Jilin University) and deposited in the herbarium of the Department of Pharmacy, Jilin University (Voucher specimen number: YQCD2014). Dulbecco's modified Eagle's medium (DMEM) was obtained from GIBCO (Grand Island, NY). Thapsigargin and 4-PBA were obtained from Sigma (St. Louis, MO). A lactate dehydrogenase (LDH) activity assay kit and CCK-8 kit were purchased from Dojindo Molecular Technologies (Tokyo, Japan). Foetal bovine serum (FBS) was purchased from HyClone (Utah, USA). An Annexin V-FITC cell apoptosis assay kit was purchased from Beyotime Biotechnology (Shanghai, China). Trypsin was purchased from Beijing Solarbio Life Sciences (Beijing, China). A ReverTra Ace qPCR RT kit was purchased from TOYOBO (Tokyo, Japan). FastStart Universal SYBR Green master mix (Rox) was purchased from Roche (Basel, Switzerland).

### 2.2. Antibodies

Rabbit polyclonal GADD153/CHOP and rabbit polyclonal GRP78/HSPA5 Abs were purchased from Novus (Colorado, USA). The mouse/rat caspase-12 affinity purified polyclonal Ab was purchased from R&D Systems (Minnesota, USA). Phospho-SAPK/JNK (Thr183/Tyr185) (81E11) rabbit and *β*-actin (13E5) rabbit mAbs were purchased from Cell Signalling Technology (Massachusetts, USA). Fluorescent secondary Abs were purchased from LI-COR Biosciences (Nebraska, USA).

### 2.3. Groupings and Treatment

H9c2 cells in the logarithmic growth phase were selected and treated in the following groups: (1) control group: H9c2 myocardial cells were cultured in a complete medium; (2) PhG-RE group: cells were treated for 24 h in a medium containing 150 *μ*g·mL^−1^ PhG-RE; (3) ERS-induced injury model group (H_2_O_2_ group): cells were treated for 18 h in a medium containing 100 *μ*mol·mL^−1^ H_2_O_2_; (4) (PhG-RE) –H_2_O_2_ group: cells were treated for 24 h in a medium containing 150 *μ*g·mL^−1^ PhG-RE and then for 18 h in 100 *μ*mol·mL^−1^ H_2_O_2_; (5) (4-PBA) –H_2_O_2_ group: cells were treated for 24 h in a culture medium containing 5 mmol·mL^−1^ 4-PBA and then for 18 h in 100 *μ*mol·mL^−1^ H_2_O_2_; (6) TG group: cells were treated for 18 h in a medium containing 50 nmol·mL^−1^ TG; and (7) (PhG-RE) −TG: cells were treated for 24 h in a medium containing 150 *μ*g·mL^−1^ PhG-RE and then for 18 h in 50 nmol·mL^−1^ TG.

### 2.4. Cell Culture

H9c2 myocardial cells were cultured in DMEM containing 10% FBS and 1% penicillin-streptomycin at 37°C in an incubator with 5% CO_2_. The complete medium was replaced every 2 d. The cells were observed under a microscope, and they grew well in a fusiform shape. When the growth density reached 70%–85%, the cells were subcultured at a 1 : 4 ratio.

### 2.5. CCK-8 Assessment of Cell Viability

H9c2 cells were microscopically examined, and the cells in the logarithmic growth phase were selected. The cell suspension was obtained through trypsin digestion and centrifuged for 5 min in a 5 mL centrifuge tube at 1000 r·min^−1^. After the residual liquid was discarded, 3 mL of DMEM complete medium was used to resuspend the cell pellets in 100 *μ*L per well, and 5.0 × 10^3^ cells were counted under the microscope. The cells were transferred to a 96-well plate and cultured in an incubator (37°C, 5% CO_2_) for 24 h to obtain completely adherent cells. The optical density (OD) was measured at a wavelength of 450 nm.

Cell viability (%) = (OD experimental group − OD blank group)/(OD control group − OD blank group) × 100%.

### 2.6. Spectrophotometric Measurement of Cell Injury

Lactate dehydrogenase (LDH) is an enzyme in the cytoplasm that is released upon cell injury, which then stably exists in the culture medium. The degree of cell injury can be determined by measuring the activity of LDH in the medium. This experiment was conducted according to the instructions for the kit. The OD was measured at a wavelength of 490 nm. There were three wells in each group.

### 2.7. Apoptosis Detection with Annexin V-FITC/PI Staining

The supernatant was removed and transferred to a flow cytometry tube. A cell suspension was prepared with adherent cells at the bottom of the trypsin digestion vessel. After the suspension was centrifuged for 6 min at 1.2 × 10^3^ r·min^−1^, the supernatant was discarded, and the cell pellets were washed thrice with PBS. This experiment was conducted according to the instructions for the Annexin V-FITC cell apoptosis kit. After the cells were cultured for 15 min at room temperature in the dark, the cells were analysed with a flow cytometer.

### 2.8. RT-qPCR Measurement of the Relative mRNA Expression of GRP78, CHOP, JNK, and Caspase-12

Total cellular RNA was extracted with a TRIzol reagent. Two microlitres of total RNA was separated by gel electrophoresis to evaluate the extraction integrity. A spectrophotometer was used to measure and calculate the OD (260)/OD (280) value. Total RNA was reverse transcribed to produce cDNA according to the instructions for the TOYOBO™ ReverTra Ace qPCR RT kit.  Table 1 shows the list of primers. According to the instructions for the Roche™ FastStart Universal SYBR Green master mix (Rox), the reaction conditions were as follows: initial denaturation at 95°C for 10 min, 95°C for 10 s, 58°C for 30 s, and 72°C for 30 s for a total of 45 cycles.

### 2.9. Western Blot Analysis of the Protein Expression of GRP78, CHOP, JNK, p-JNK, and Caspase-12

Protein lysate (50 *μ*g) was separated by electrophoresis on a 10% separating gel, followed by transfer to a PVDF membrane. The PVDF membrane was blocked in the TBS-T solution containing 5% skimmed milk at room temperature for 90 min. The blocked PVDF membrane was incubated overnight with antibodies 1, 2, 3, and 4 at 4°C, and then for 90 min with goat anti-rabbit IgG (1 : 1000) at room temperature. Finally, the membrane was developed with a two-colour infrared laser imaging system.

### 2.10. Statistical and Analytical Methods

The data in this study were processed with SPSS 24.0 software, and the results are presented as the means ± SD. The data from multiple groups were compared with ANOVA, while the data from two groups were compared with *t*-tests. A value of *P* < 0.05 was considered to have statistical significance. ImageJ software was used to quantitatively analyse the greyscale values of the protein bands.

## 3. Results

### 3.1. Influence of PhG-RE on Cell Viability

**Figure 1:**
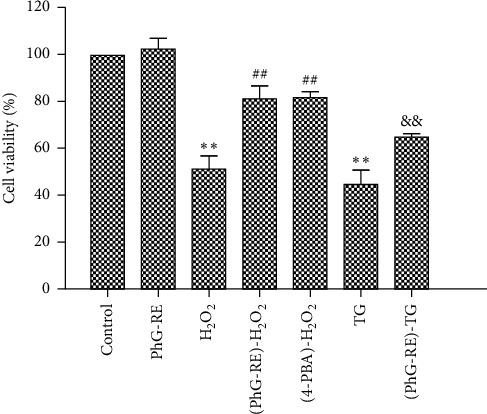
Cell viability among the different groups. Drug treatment for each group according to the experimental design. Cell viability was measured by the CCK-8 assay. Note: compared with the control group, ^*∗∗*^*P* < 0.01; compared with the H_2_O_2_ group, ^##^*P* < 0.01; and compared with the TG group, ^&&^*P* < 0.01.

As shown in Figure 1, compared to that of the control group, the viability of H9c2 cells in the PhG-RE group did not change significantly (*P* > 0.05), but the viability of cells in both the H_2_O_2_ and TG groups decreased significantly. Compared to that of the H_2_O_2_ group, the viability of cells in both the (PhG-RE)-H_2_O_2_ and the (4-PBA)-H_2_O_2_ groups increased significantly. Compared to that of the TG group, cell viability in the (PhG-RE)-TG group increased significantly. All of the results were statistically significant (*P* < 0.01).

### 3.2. Influence of PhG-RE on Cell Injury

**Figure 2:**
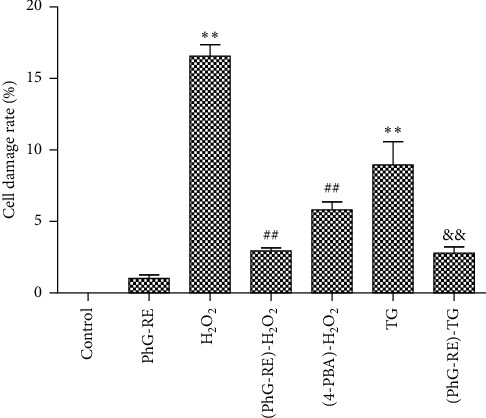
The effect of PhG-RE on the release of LDH from H9c2 cells. Drug treatment for each group according to the experimental design. The release of LDH was measured using an LDH kit. Compared with the control group, ^*∗∗*^*P* < 0.01; compared with the H_2_O_2_ group, ^##^*P* < 0.01; and compared with the TG group, ^&&^*P* < 0.01.

As shown in Figure 2, although the cells in the PhG-RE group were damaged compared to those in the control group, that damage did not reach statistical significance (*P* > 0.05). Cell injury in the H_2_O_2_ and TG groups increased significantly. Compared to that of the H_2_O_2_ group, cell injury in the (PhG-RE)-H_2_O_2_ and (4-PBA)-H_2_O_2_ groups decreased significantly (*P* < 0.01). Compared to that of the TG group, cell injury in the (PhG-RE)-TG group decreased significantly (*P* < 0.01).

### 3.3. Influence of PhG-RE on Cell Apoptosis

**Figure 3:**
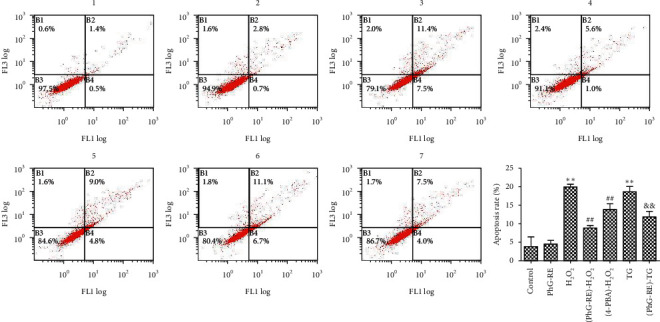
The effect of PhG-RE on H9c2 cell apoptosis, as detected by Annexin V and PI double-staining. Drug treatment for each group according to the experimental design. All groups were stained with Annexin V and PI, and then analysed by flow cytometry. 1: Control group, 2: PhG-RE group, 3: H_2_O_2_ group, 4: (PhG-RE)- H_2_O_2_ group, 5: (4-PBA)- H_2_O_2_ group, 6: TG group, and 7: (PhG-RE)-TG group. ^*∗∗*^*P* < 0.01, compared with the control group; ^##^*P* < 0.01, compared with the H_2_O_2_ group; and ^&&^*P* < 0.01, compared with the TG group.

As shown in Figure 3, compared to that of the control group, cell apoptosis in the PhG-RE group did not change significantly (*P* > 0.05), while that in the H_2_O_2_ and TG groups increased significantly (*P* < 0.01). Compared to that of the H_2_O_2_ group, cell apoptosis in the (PhG-RE)-H_2_O_2_ and (4-PBA)-H_2_O_2_ groups decreased significantly (*P* < 0.01). Compared to that of the TG group, cell apoptosis in the (PhG-RE)-TG group decreased significantly (*P* < 0.01).

### 3.4. Influence of PhG-RE on the Expression of GRP78, CHOP, JNK, and Caspase-12 mRNA

**Figure 4:**
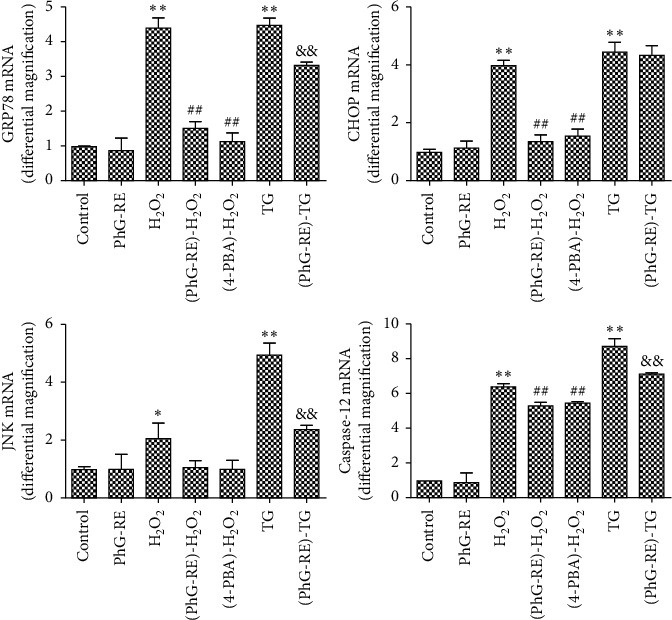
The effect of PhG-RE on GRP78, CHOP, JNK and Caspase-12 mRNA expression in H9c2 cells, as determined by RT-qPCR. The data were normalized to *β*-actin levels. Relative fold changes in the expression levels of target genes in all groups were determined using the 2^−∆∆Ct^ method. Compared with the control group, ^*∗∗*^*P* < 0.01, ^*∗*^*P* < 0.05; compared with the H_2_O_2_ group, ^##^*P* < 0.01; and compared with the TG group, ^&&^*P* < 0.01.

The relative mRNA expression in each experimental group was quantitatively measured with RT-qPCR. As shown in Figure 4, compared to that of the control group, the relative mRNA expression of GRP78, CHOP, and Caspase-12 increased significantly (*P* < 0.01) in the H_2_O_2_ group, and the relative mRNA expression of JNK also increased (*P* < 0.05). In the TG group, the relative mRNA expression of GRP78, CHOP, JNK, and Caspase-12 increased significantly (*P* < 0.01). Compared to that of the H_2_O_2_ group, the relative mRNA expression of GRP78, CHOP, and Caspase-12 in the (PhG-RE)-H_2_O_2_ and (4-PBA)-H_2_O_2_ groups decreased significantly (*P* < 0.01), while the relative mRNA expression of JNK did not change significantly (*P* > 0.05). Compared to that of the TG group, the relative mRNA expression of GRP78, JNK, and Caspase-12 in the (PhG-RE)-TG group decreased (*P* < 0.01), and the relative mRNA expression of CHOP did not change significantly (*P* > 0.05).

### 3.5. Influence of PhG-RE on the Protein Expression of GRP78, CHOP, and Caspase-12

**Figure 5:**
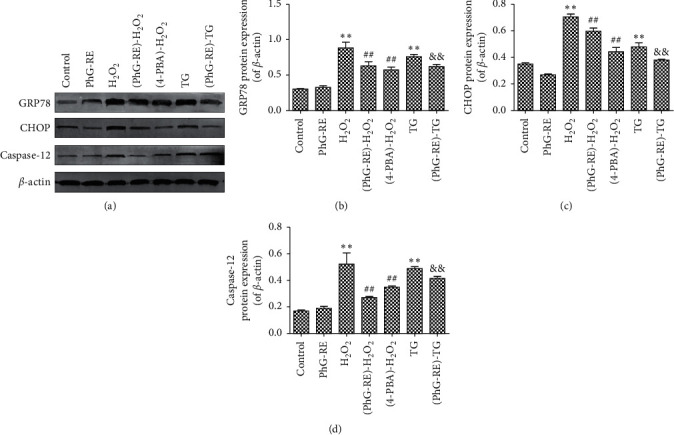
The effect of PhG-RE on GRP78, CHOP, and Caspase-12 protein expression in H9c2 cells. (a). Representative western blots showing GRP78, CHOP, Caspase-12, and *β*-actin expression; (b). Quantitative analysis of GRP78 expression. (c). Quantitative analysis of CHOP expression. (d). Quantitative analysis of Caspase-12 expression. ^*∗∗*^*P* < 0.01 vs. the control group; ^##^*P* < 0.01 vs. the H_2_O_2_ group; and ^&&^*P* < 0.01 vs. the TG group.

Figure 5 shows the protein expression levels of GRP78, CHOP, and Caspase-12. Compared to that of the control group, the expression of these three proteins in the H_2_O_2_ and TG groups increased significantly after the cells were treated (*P* < 0.01). Compared to that of the H_2_O_2_ group, the expression of GRP78, CHOP, and Caspase-12 decreased significantly in the (PhG-RE)-H_2_O_2_ and (4-PBA)-H_2_O_2_ groups (*P* < 0.01). Compared to that of the TG group, the expression of GRP78, CHOP, and Caspase-12 in the (PhG-RE)-TG group decreased significantly.

### 3.6. Influence of PhG-RE on JNK and p-JNK

**Figure 6:**
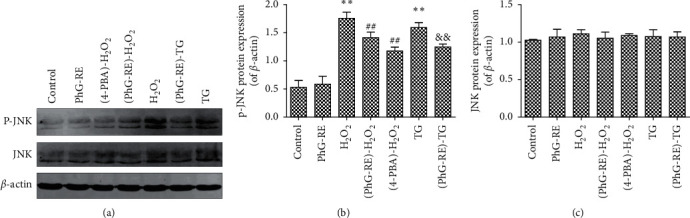
The effect of PhG-RE on JNK and p-JNK protein expression in H9c2 cells. (a). Representative western blots showing JNK and p-JNK expression. (b). Quantitative analysis of p-JNK expression. (c). Quantitative analysis of JNK expression. ^*∗∗*^*P* < 0.01 vs. the control group; ^##^*P* < 0.01 vs. the H_2_O_2_ group; and ^&&^*P* < 0.01 vs. the TG group.

As shown in Figure 6, compared to that of the control group, the expression of p-JNK in the H_2_O_2_ and TG groups increased significantly (*P* < 0.01). Compared to that of the H_2_O_2_ group, the expression of p-JNK in the (PhG-RE)-H_2_O_2_ and (4-PBA)-H_2_O_2_ groups decreased significantly (*P* < 0.01). Compared to that of the TG group, the expression of p-JNK in the (PhG-RE)-TG group decreased significantly (*P* < 0.01). Concerning the expression of JNK, there were no significant changes in any of the experimental groups (*P* > 0.05).

## 4. Discussion

Cardiovascular diseases, such as I/R injury, can induce ERS through oxidative stress, calcium overload, and the formation of oxygen-free radicals. Under these conditions, the ER fails to correctly fold and process proteins, leading to the accumulation of misfolded or unfolded proteins in the endoplasmic reticulum, which then induces the unfolded protein response (UPR). The UPR is a cellular stress response that initially inhibits apoptosis, but ultimately accelerates it.

Studies have confirmed that ERS partly accounts for why ischaemia and anoxia can exacerbate heart disease [6, 7]. The apoptotic factors, GRP78, Caspase 12, CHOP, and JNK, are important participants in the occurrence of disease [8–11]. In this study, we researched the antagonism and related mechanisms of PhG-RE in H9c2 cell apoptosis of H_2_O_2_-induced ERS. Using 100 *μ*mol·mL^−1^ H_2_O_2_ as an inducer, an ERS model was established in H9c2 cells. To ensure validity and accuracy of the model, the specific endoplasmic reticulum stress activator thapsigargin (TG) was used to determine whether PhG-RE protects cells against ERS-induced apoptosis. The ERS inhibitor 4-PBA was also used to determine whether PhG-RE produces an effect similar to that of 4-PBA in effectively countering the apoptosis caused by the H_2_O_2_-induced ERS.

In this study, both H_2_O_2_ and the ERS activator TG caused cell apoptosis by mediating ERS in cells. LDH, a stable enzyme present in the cytoplasm, leaves the cell as cell rupture occurs. Therefore, cellular damage can be determined by measuring LDH leakage in the medium. The cell viability and LDH leakage represent the level of damage to H9c2 cells and can be used to evaluate the protective effect of PhG-RE pre-treatment on cells. Exposure to PhG-RE effectively reduced cell apoptosis induced by TG and H_2_O_2_, enhanced survival, and reduced LDH release. This finding shows that PhG-RE plays a role similar to that of 4-PBA in protecting cells. This result indicates that PhG-RE can protect cells against ERS-induced injury and apoptosis.

In normal cells that are not under stress, GRP78, which is associated with ERS, binds with multiple ER transmembrane proteins on the cell surface and is in an inactive and stable state. When ERS first occurs, unfolded proteins induce the UPR, which promotes the dissociation of transmembrane proteins from GRP78. After dissociation, GRP78 binds with unfolded or misfolded proteins to counter ERS through an increased protein expression [12–14]. As ERS progresses, the function of the endoplasmic reticulum is severely damaged, and the UPR initiates apoptosis by transcribing and activating the CCAAT/EPB homologous protein (CHOP), the c-Jun N-terminal kinase (c-JNK), and Caspase-12. In normal cells, the expression of CHOP is low and is primarily regulated at the transcriptional level, and it has been shown that ERS and the UPR induce CHOP transcription [15]. When ERS induces apoptosis, the expression of CHOP and its accumulation in the cell nucleus are increased [16]. In addition, due to the pro-apoptotic effects of the UPR, pro-caspase-12 is released, and apoptosis is initiated. The released pro-caspase-12 is cleaved into its active form, Caspase-12. An important reason for ERS initiating apoptosis is that Caspase-12 is activated by ERS [17]. JNK, another element that induces apoptosis, is activated by the IRE-1-TRAF2-ASK1 complex, which is generated during apoptosis. The downstream Caspase family, which plays a pro-apoptotic role, is also activated.

**Figure 7:**
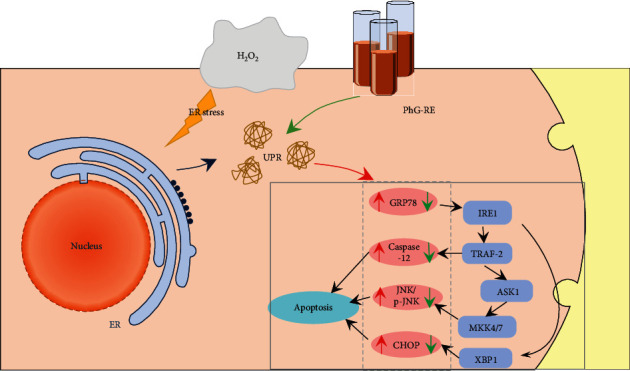
PhG-RE reduces apoptosis by inhibiting the signal expression of apoptosis-related pathways in ERS.

Drawing upon gene and protein detection technology, this study examined the mechanism by which PhG-RE protects the myocardial cells. When ER stress occurs, the transcription factor CHOP can cause the release of Cyt C and apoptosis-inducing factors (AIFs), which results in cellular apoptosis [18–20]. Moreover, the highly conserved ER membrane protein IRE1*α* can be phosphorylated and induce cell apoptosis by activating an alternative pathway through TRAF2- and apoptosis signal-regulating kinase 1 (ASK1)-mediated signal transduction [21]. Pre-treatment with PhG-RE reduced the mRNA expression of GRP78, CHOP, and Caspase-12 during ER stress in H_2_O_2_-induced cells, as well as the TG-induced mRNA expression of GRP78, JNK, and Caspase-12. PhG-RE pre-treatment had an antagonistic effect on the H_2_O_2_- and TG-induced increases in GRP78, CHOP, p-JNK, and Caspase-12 protein expression levels. These results show that PhG-RE plays a role similar to that of the ERS inhibitor 4-PBA in protecting myocardial cells and countering ERS-induced apoptosis by reducing the mRNA and protein expression of factors associated with ERS-induced apoptosis. Although there was no significant change in the JNK protein, this finding is consistent with the literature showing that JNK functions through its activated form, p-JNK. The diagram in Figure 7 shows the signalling pathway, which may clearly explain how PhG-RE works to protect the myocardial cells against ERS-induced apoptosis.

## 5. Conclusions

Myocardial ischaemia-reperfusion injury is the main cause of exacerbated myocardial injury. As another factor that induces myocardial disease, ERS has been a new focus of research that aims to improve the effects and efficacy of myocardial disease treatment. This study further explored the role of PhG-RE in protecting myocardial cells and showed that PhG-RE-mediated myocardial protection was strongly related to the regulation of ERS-induced apoptosis. However, as myocardial disease is caused by multiple factors, further studies will need to be performed on the effects of PhG-RE.

## Figures and Tables

**Table 1:** Primer sequence for RT-qPCR.

Gene	Primer sequence (5′-3′)	Primer sequence (5′-3′)	Annealing temperature (°C)	Gene registration number	prodSize (bp)
GRP78	F:AGGATGTAGGCACGGTGGCT	R:GCCACATACGACGGTGTGAA	56	25617	127
JNK	F:AGCCAGTCAGCCGAGAGATT	R:GGTGCTGGACAGCTTCGTCT	56	116554	86
CHOP	F:CCTCGCTCGCCAGATTCCAG	R:CCTACTCCTTCATGCGCTGT	56	29467	133
Caspase-12	F:TCGGAGAAGCAGCGAGCTTA	R:TTGGTAATGTGACCTGCAA	56	156117	106
*β*-actin	F:TCATGAAGTGTGACGTTGACATCT	R:CCTAGAAGCATTTGCGGTGCACTG	56	81822	165

## Data Availability

The data used to support the findings of this study are available from the corresponding author upon request.
